# Negativity about the outcomes of extreme prematurity a persistent problem - a survey of health care professionals across the North Queensland region

**DOI:** 10.1186/s40748-020-00116-0

**Published:** 2020-04-28

**Authors:** Susan Ireland, Sarah Larkins, Robin Ray, Lynn Woodward

**Affiliations:** 1 The neonatal unit, Townsville University Hospital, Angus Smith Drive, Douglas, Queensland 4814 Australia; 2 Department Medicine and Dentistry, James Cook University, Townsville, Australia

**Keywords:** Extreme prematurity, Attitudes, Outcomes, Resuscitation, Decision-making, Mortality, Morbidity

## Abstract

**Background:**

Extremely preterm babies are at risk of significant mortality and morbidity due to their physiological immaturity. At periviable gestations decisions may be made to either provide resuscitation and intensive care or palliation based on assessment of the outlook for the baby and the parental preferences. Health care professionals (HCP) who counsel parents will influence decision making depending on their individual perceptions of the outcome for the baby. This paper aims to explore the knowledge and attitudes towards extremely preterm babies of HCP who care for women in pregnancy in a tertiary, regional and remote setting in North Queensland.

**Methods:**

A cross sectional electronic survey of HCP was performed. Perceptions of survival, severe disability and intact survival data were collected for each gestational age from 22 to 27 completed weeks gestation. Free text comment enabled qualitative content analysis.

**Results:**

Almost all 113 HCP participants were more pessimistic than the actual outcome data suggests. HCP caring for women antenatally were the most pessimistic for survival (*p* = 0.03 at 23 weeks, *p* = 0.02 at 25,26 and 27 weeks), severe disability (*p* = 0.01 at 24 weeks) and healthy outcomes (p = 0.01 at 24 weeks), whilst those working in regional and remote centres were more negative than those in tertiary unit for survival (*p* = 0.03 at 23,24,25 weeks). Perception became less negative as gestational age increased.

**Conclusion:**

Pessimism of HCP may be negatively influencing decision making and will negatively affect the way in which parents perceive the chances of a healthy outcome for their offspring.

## Introduction

Delivery of an extremely premature infant below 28 weeks completed gestation is uncommon, affecting less than 1 % of babies born in Australia [1]. Depending on the jurisdiction, a ‘grey zone’ exists between 23 and 25 weeks completed gestation where the risk of death or significant disability necessitates careful thought between the provision of intensive care or the option of palliation for these infants, and resuscitation below 23 weeks is usually discouraged [2–5]. The decision to provide intensive care requires consensus between the treating teams and the parents of the baby, and health care professionals (HCP) provide counselling to the parents prior to decision making. Few parents who face early delivery have adequate medical knowledge to enable them to make any decisions alone, therefore the knowledge of the HCP about both the potential outcomes in terms of death and disability for the baby is essential during counselling [5]. Where a decision is made to provide active care, obstetric care including antenatal steroids and magnesium sulphate administered to the mother, as well as consideration of operative delivery for babies in distress may improve the prospects of healthy survival [6, 7].

With technological advances and enhanced quality of care, the outlook for these vulnerable babies is improving over time [8] and thus HCPs need an awareness of contemporaneous and locally relevant data. In addition, parental requests for the provision of active care for babies from 22 weeks completed gestation are recognised in Australia [9] and elsewhere [10, 11]. HCPs will therefore also potentially need an approach to address this parental demand.

Early Australian studies on the knowledge and attitudes of HCP focussed primarily on the tertiary obstetrician and neonatologists [12–14]. However, it is now acknowledged that a wider range of HCPs may also influence parental decision making including midwifery staff and neonatal nurses as well as clinicians involved in care prior to transfer to a tertiary hospital [15, 16]. These studies suggest that HCPs tend to be negative and have a lower expectation of both survival and morbidity than is the case, with obstetricians being the most negative and neonatologists more optimistic. A more recent study [16] included obstetric and midwifery staff at level 1 and 2 hospitals but no junior obstetric staff, and the neonatology staff of the retrieval service. This study suggested clinicians continued to overestimate rates of adverse outcomes. Message framing will influence parental decision making and outlook, and clinicians with negative perceptions are likely to both convey this to the parents [17] and manage the pregnancy and baby accordingly [18, 19]. Individual clinician personality and bias towards poor outcomes will also effect message framing [19].

Parents of extremely premature babies who are beyond the ‘grey area’ of decision making will also need accurate information and consistency from HCP about the potential outcome for their child, as extreme prematurity will have a considerable impact on the parents’ future lives [20], particularly where the care is often provided far from the family home.

This study aims to investigate the knowledge of HCP and ascertain their attitudes towards the provision of care for extremely premature babies, including which factors staff feel should be considered when offering, or not offering, intensive care in North Queensland.

## Methods and analysis

A cross-sectional electronic survey of HCPs was administered on the SurveyMonkey platform (SurveyMonkey Inc. Ca. U.S.A.). HCP at three centres in North Queensland were invited to participate.

The study centres include the largest provider of tertiary neonatal care in Northern Australia, one of two regional referral centres and a remote hospital. The tertiary hospital provides care for babies of all gestations and offers care for babies with surgical and medical conditions. It cares for all babies who receive neonatal intensive care below 28 weeks gestation in North Queensland. More than half the parents delivering extremely preterm babies reside within other health districts, and nearly a quarter are retrieved following delivery at smaller health care facilities [21]. The regional referral hospital is a regional hospital that offers care for babies over 32 weeks gestation, whist the other referral hospital is a small remote centre which can offer only low acuity care to babies over 32 weeks gestation. The three sites were chosen as they represent the range of hospitals staffed by resident obstetric and paediatric services. The non-tertiary sites often need to refer women with vulnerable pregnancies to the tertiary hospital for care but will be required to provide initial care to periviable babies who cannot be transferred to the tertiary units in-utero.

Following identification of a pregnancy at risk of extreme prematurity, parents are counselled by senior obstetric and neonatal staff, including potential outcomes and the expected neonatal course. Those pregnancies in the ‘grey zone’ are identified, and options to provide full intensive care or palliation are discussed. Parents are also given the option to initiate full resuscitation, with the option of redirecting care either during resuscitation or on the neonatal unit, where the baby is in poor condition or appears to be significantly compromised. Where there is potential for resuscitation, obstetric actions to optimise the condition of the baby are initiated. Decisions to resuscitate often involve several discussions, during which time the woman will be cared for by midwifery staff, and the neonatal unit is toured together with a neonatal nurse. Where the family is from a peripheral centre, often only brief counselling is given prior to transfer and tertiary obstetrician review.

### Survey design

The survey was designed with questions about the demographics of the respondent including primary location of work, work stream, experience, social contact with people with disability and whether their religious beliefs influenced their decision making. Respondents were asked: i) whether they cared for pregnant women under 28 weeks gestation who were at risk of premature delivery: ii) if they had ever been asked by a parent for their personal opinion about whether a baby should receive intensive care or palliative care: and iii) their confidence in discussing extreme prematurity with patients. Further questions explored their knowledge of rates of: i) survival: ii) severe disability: and iii) intact survival at different gestations from 22 to 27 weeks completed weeks gestation. Replies to survival and outcomes were given as one of five quintiles in 20% divisions as it was considered less intimidating to participants than asking for exact estimates, whilst still being accurate enough for analysis. Participants were asked to rank their opinion about other factors which may influence the decision to offer intensive care to extremely preterm babies, and give an opinion about the most appropriate gestation from which intensive care should be offered to premature babies, at which gestation parents could be sole decision makers, whether staff could override parents’ wishes, and the gestation at which the participant would want a potential extremely premature baby of their own to be resuscitated. Free text was allowed for participants to expand on their replies. Although similar studies are found in the literature, the questionnaire was not based specifically on any of these as none captured all the data of interest. All gestations of babies from 22 to 27 completed weeks were included although resuscitation is usually provided at the older gestations.

The survey was piloted with a group of senior nursing and medical staff and a psychologist involved in neonatal care to assess face validity and adapted to ensure clarity.

### Participant recruitment

An email link was sent by the primary investigator to all neonatal, paediatric and obstetric medical staff specialist or doctors on college training programs at the tertiary centre. Senior nursing managers sent the link to registered midwives and neonatal intensive care nurses at the tertiary centre and a research co-ordinator at each of the smaller centres sent the link to obstetric, midwifery and paediatric staff. A second email was sent two weeks later to promote participation. It was not possible to identify which staff had responded to the link, beyond the demographic data related to work stream.

### Data analysis

The survey data were imported directly from the survey tool and were analysed using IBM SPSS 25 (Armonk, NY, USA). Analysis used frequencies for numerical data. Chi square was used for categorical variables. Where categorical data with multiple ordinal responses occurred, Kruskal-Wallis H test to compare means was utilised. Significance was defined as *p* < 0.05. A comparison was made between HCP who care for women primarily prior to delivery - obstetrics and midwifery staff (referred to as antenatal HCP), and after delivery – neonatologists, neonatal nurses and paediatricians (who were included as they provide counselling at the non-tertiary centres and at the tertiary centre provide neonatal care on the postnatal wards), referred to as postnatal HCP. Questions about factors which may influence opinions positively or negatively towards resuscitation were given as a Likart score, with scores of very likely and likely to imply a positive influence to offer intensive care, a score of neutral was considered to indicate that the factor was not contributory to the opinion, whilst an unlikely or very unlikely score was considered to indicate the factor would make the HCP less likely to agree with resuscitation. Missing data was excluded from analysis. Content analysis was performed on the qualitative data using a process of coding for thematic classification.

### Comparison data

The tertiary unit studied had outcomes for survival and all short-term morbidities within the expected range for units within the Australian and New Zealand Neonatal Network (ANZNN). The ANZNN data collection is a collaborative network established under the recommendation of the National Health and Research Councils Expert Panel on Perinatal Morbidity [22]. For this study, data from the tertiary unit database for the years 2013 to 2017 inclusive have been used for survival. Long term follow-up for babies born from 2011 to 2014 inclusive were considered. Follow up data for the tertiary unit are around 50% for all gestations due to difficulty in getting patients long term data from outside the district. The data given in the ANZNN comparative database suggests that outcomes for severe disability for the tertiary unit compares positively to the mean for the ANZNN group. The mean rates for severe disability and typical development for the ANZNN have been used for expected long term outcomes because of concerns that the lower follow up rate of the tertiary unit might be a source of positive bias where more regional and remote children are excluded.

### Ethical approval

The study was approved by the Townsville hospital human research ethics committee HREC/15/QTHS/194, and acknowledged by James Cook University (JCU) ref. 6485. Governance approval was given by all participating sites and JCU

## Results

### Participants

E-mails were sent to 174 potential participants, with 113 replies (total response rate 64.9%). Demographic details are shown in Table 1. Not all participants answered all questions.

**Table 1:** Demographics of respondents to survey n = number of respondents

Location – respondents at each site/number invited to participate *n* = 113	Tertiary centre	74/116 (64%)
	Regional centre	17/30 (57%)
	Remote centre	22/28 (79%)
Work stream *n* = 112	Midwifery	41 (36.3%)
	Obstetrics	17 (15.0%)
	Neonatal nurse	28 (24.8%)
	Neonatologist	5 (4.4%)
	Paediatrician	21 (28.6%)
Contact with women at risk of extreme prematurity n = 113	Yes	104 (92.0%)
Duration of work experience in years n = 112	< 1	11 (9.7%)
	1–5	27 (23.9%)
	> 5–9	24 (21.2%)
	10+	50 (44.2%)
Confidence in knowledge of implications of extreme prematurity *n* = 112	Not Confident	30 (26.8%)
	Neutral	17 (15.0%)
	Confident	65 (58.0%)
Ever asked for personal opinion about resuscitation by a woman at risk of extreme prematurity (numbers asked/total respondents) *n* = 110	Midwifery	17/41 (42%)
	Obstetrics	13/17 (77%)
	Neonatologist	4/5 (80%)
	Neonatal nurse	13/28 (46%)
	Paediatrician	11/21 (52%)

**Fig. 1:**
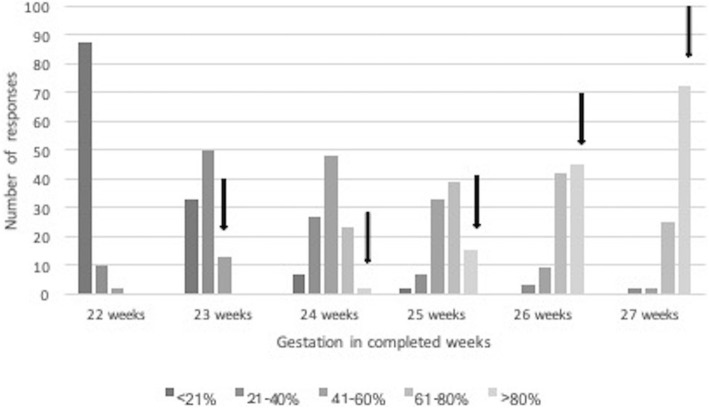
Estimates of survival at different completed weeks gestation, with responses given in quintiles. Accurate survival figures represented by the solid arrow indicating actual survival quintile based on data for the tertiary unit for the years 2013 to 2017 inclusive. Responses to the left of the arrow indicate a negative understanding of the survival rates for each gestation. Data not given for 22 week gestation babies as the numbers treated were small

**Fig. 2:**
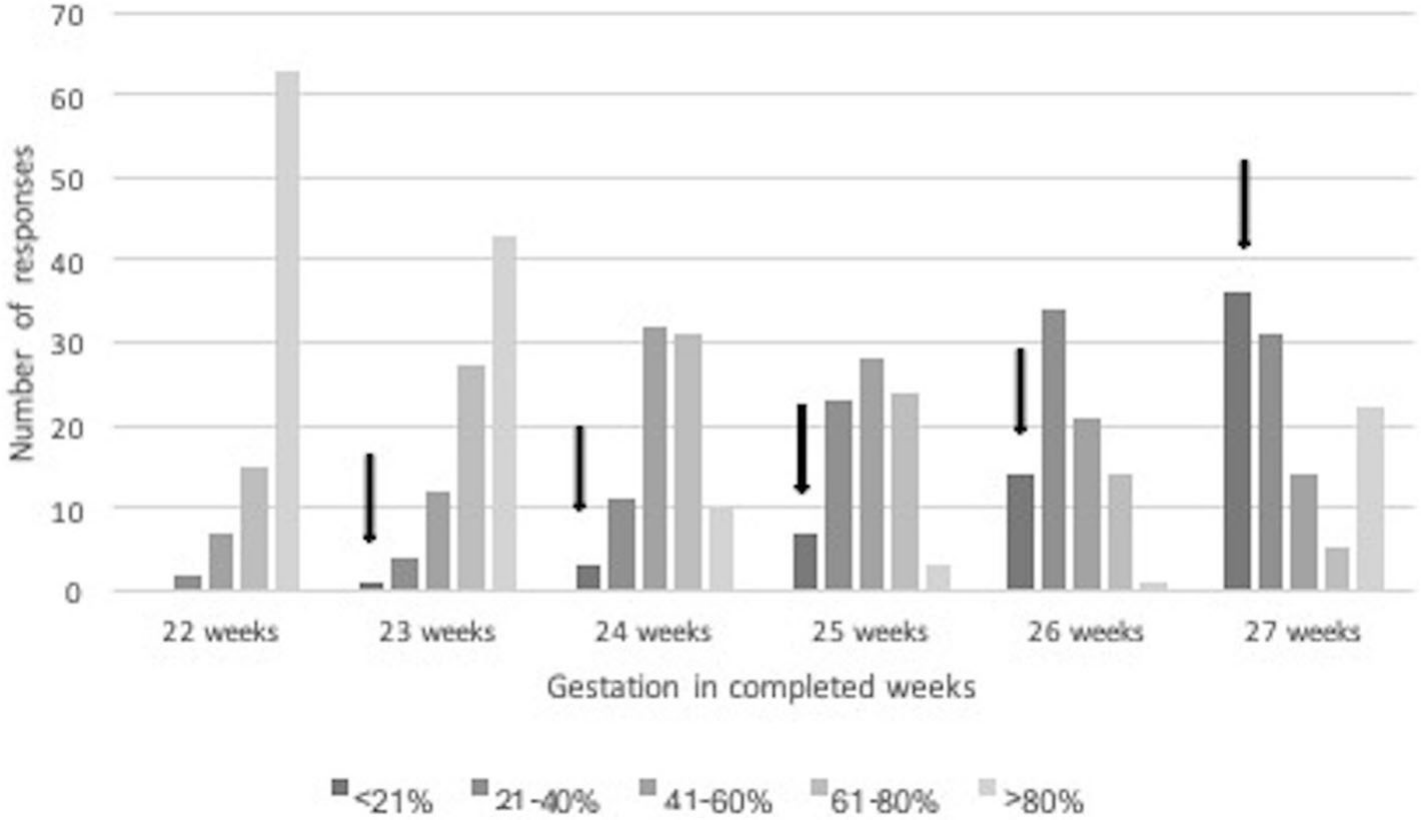
Estimates of severe disability in quintiles given by participants. The quintile based on ANZNN data for babies born from 2011 to 2014 inclusive is represented by the solid arrow. All responses to the right of the arrow represent negative estimates of severe disability. Data for 22 week gestation babies is not given in the ANZNN database

**Fig. 3:**
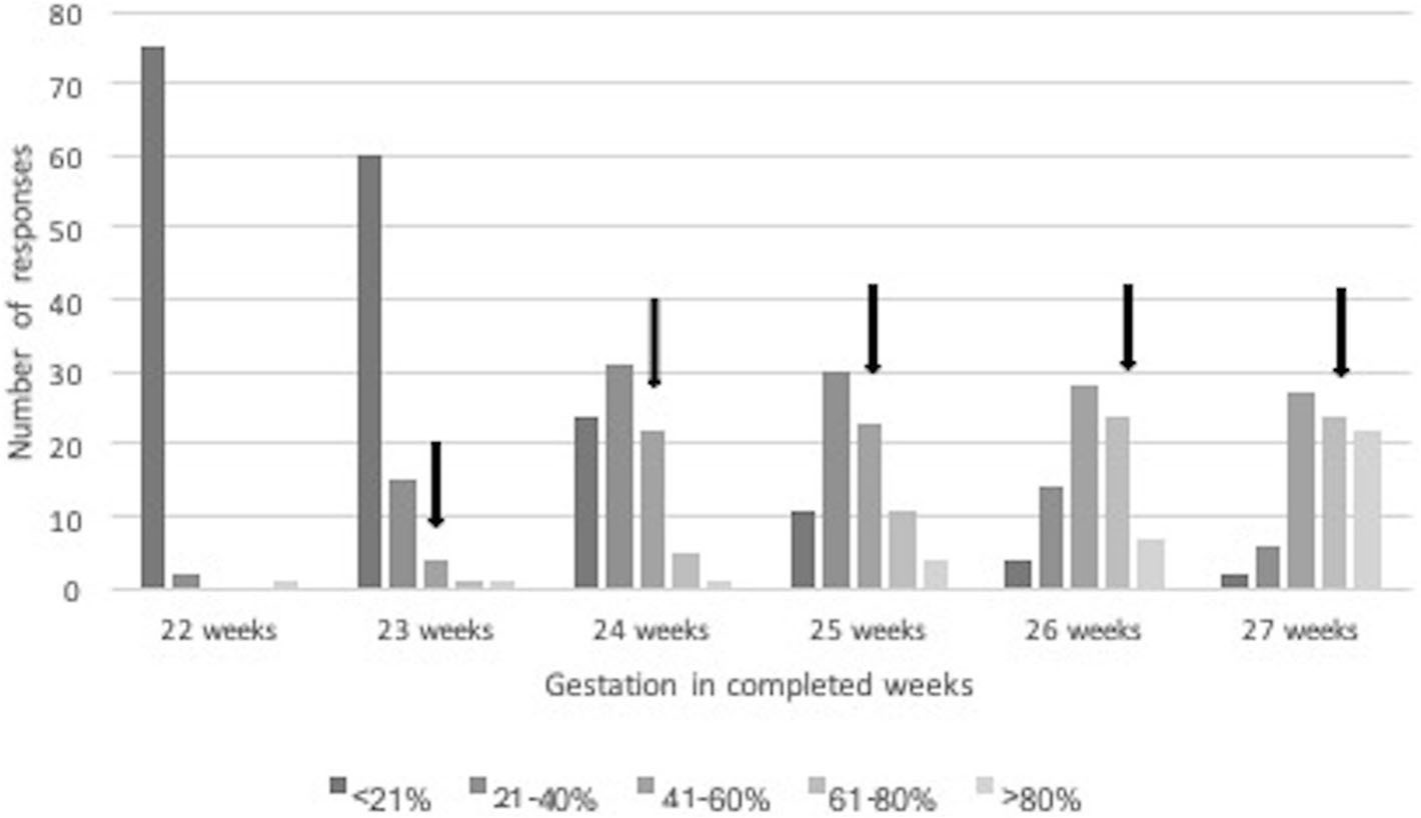
Estimates of rates of intact survival in quintiles. Actual rates of typical development as given by the ANZNN database for 2011 to 2014 inclusive are indicated by the solid arrow. Responses to the left of the arrow for each gestation indicate a negative response. Accurate data omitted for 22 completed weeks gestation as data may be inaccurate because of small numbers of survivors within the group and is not given in the ANZNN database

Some participants did not complete all aspects of the survey – with midwives and those from outside of the tertiary centre less likely to answer all questions. For different gestations, 81–91% of antenatal HCP, and 90–98% post-delivery HCP answered survival questions, 64–72% of antenatal HCP and 83–90% postnatal HCP answered severe disability questions, 59–67% of antenatal HCP and 77–87% postnatal HCP answered questions about intact survival. There was no clear pattern in gestational age for the missing data. Survival questions were answered by 89–97% by the tertiary group and 79–90% by the non-tertiary participants, Severe disability questions were answered by 78–84% by the tertiary group and 62–74% by the non-tertiary group, and the intact survival questions answered by 69–81% by the tertiary and 56–69% of the non-tertiary group (Figs. 1, 2 and 3).

Whilst 92% of the HCP had contact with women at risk of extreme premature delivery, only 52.8% had been asked for their advice about the resuscitation of a baby. Over half of the study group had personal contact with a person with severe disability, but few acknowledged religious beliefs shaping their opinions. Almost all the neonatologists and obstetricians had been asked for their personal opinions by patients about whether the parent should opt for active care. Excluding them, there were no significant differences between work streams, location or level of experience for being asked an opinion about intensive care provision, or with confidence in knowledge.

Participants were asked to indicate whether specific factors would positively or negatively influence their propensity to offer intensive care to extremely preterm babies (Table 2).

**Table 2:** Factors which might influence HCP to be more likely (positive influence) or less likely (negative influence) to consider intensive care to be appropriate

	Negative influence	Neutral	Positive influence
Parents request intensive care, clinician feels it is not in babys best interest *n* = 97	21 (21.6%)	9 (9.3%)	67 (69.1%)
Clinician promotes intensive care where parent does not wish provision of NICU *n* = 96	36 (37.5%)	21 (21.9%)	39 (40.6%)
Low socio-economic family *n* = 97	4 (4.1%)	87 (89.7%)	6 (6.2%)
Mother under 20 years of age *n* = 97	2 (2.1%)	89 (91.8%)	6 (6.2%)
Mother over 40 years of age *n* = 97	1 (1.0%)	85 (87.6%)	11 (11.3%)
Children in state care n = 97	15 (15.5%)	76 (78.4%)	6 (6.2%)
Known surgical anomaly usually provided care at term *n* = 97	58 (59.8%)	30 (30.9%)	9 (9.3%)
Known trisomy 21 *n* = 97	54 (55.7%)	35 (36.1%)	7 (7.2%)
Previous pregnancy loss n = 97	1 (1.0%)	63 (64.9%)	33 (34.0%)
No live children n = 97	2 (2.1%)	61 (62.9%)	34 (35.1%)

The gestational age at which the participant would offer NICU to a patient was significantly lower than the gestation at which HCP would choose for themselves. HCP considered that 24 weeks (IQR 24–25) was an appropriate lowest gestation to offer parents, with midwifery and paediatric staff considering 25 weeks (IQR 24–26) and obstetricians and neonatal nurses choosing 26 weeks (IQR 25–26 and 24–26 weeks respectively). There were insufficient neonatologist response to analyse. For all HCP, a choice from a gestational age of 25 (IQR 24–26) compared to offer for patient 24 (IQR 24–25) was significantly different *p* = 0.00.

Comparison was made between the antenatal HCP (58 participants) and the HCP caring for the baby after delivery (53 participants). Analysis showed a significant difference in the perception for survival for most gestations from 23 to 27 completed weeks; 23 weeks p = 0.03 (χ^2^ [1] 4.64), 25 weeks *p* = 0.02 (χ^2^ [1] 4.49), 26 weeks p = 0.02 (χ^2^ [1] 5.05), 27 weeks p = 0.02 (χ^2^ [1] 6.76), as well as significant differences in perception for severe disability at 24 weeks *p* = 0.01 (χ^2^ [1] 4.64), and intact survival p = 0.01 (χ^2^ [1] 7.35), with the antenatal HCP more negative for each parameter.

Analysis of tertiary hospital HCP (74 participants) compared to regional and remote HCP (39 participants) showed that the regional and remote HCP were significantly less optimistic about survival at 23 weeks *p* = 0.03 (χ^2^ [1] 5.07), 24 weeks p = 0.03 (χ^2^ [1] 5.13), and 25 weeks p = 0.03 (χ^2^ [1] 3.95), but there were no other significant differences for estimates of severe disability or healthy outcomes.

Participants were asked at which gestation parents should be the final decision maker (Table 3).

**Table 3:** HCP opinion about the gestation at which they considered that parents could be the final decision makers for decisions about care. Data expressed in numbers (percent) (* signifies significant *p*=<0.05)

	Informed parent can make final decision *n* = 83	Clinician can make a final decision regardless of parental preference *n* = 82	*P* value
Never	32 (38.6%)	13 (15.9%)	0.01*
< 25 weeks	45 (54.2%)	53 (64.6%)	0.47
25–28 weeks	6 (7.2%)	16 (19.5%)	0.04*

* denotes significant finding *p*=<0.05

One hundred and twenty free text comments were received. These were divided into six themes (Table 4).

**Table 4:** Themes and representative quotes for content analysis of the free text

Theme	Representative quotation
Every situation is different	The decision should be individualised for every family (Paediatrician)
The burden of guilt is too much for parents	No parent wants to live with the ‘did I kill my baby’ dilemma (Neonatal nurse)
Parental choice is paramount	Will the parents be willing to look after a disabled child they didn’t want resuscitated? (Midwife) Parents are influenced by lesser degrees of disability and not only severe disability (Obstetrician)
Advocating for the baby	At 24 weeks approximately half the survivors will have only mild or no disability. The uncertainty of outcome combined with uncertainty around exact gestation make any definitive advice around outcome imprecise. Resuscitation is not the last opportunity to withhold treatment from a baby … Choosing death is not necessarily a decision to be rushed. The disabled have rights. (Neonatologist) If a healthcare professional believes the chance of survival for an infant is good, full active management should happen regardless of the parental opinion. I believe we have to advocate for the baby when the parents do not have its best interests in mind. (Midwife)
Following the law	When it comes to the wellbeing of a premature infant, there are legal guidelines regarding viability to protect the unborn child (Nurse)
Ways to educate pregnant women about prematurity	Perhaps a basic handout of survival and disability statistics of babies born less than 30 weeks gestation should be given to parents at their first booking-in clinic. If the parents have a basic awareness, they may already have made a decision should they be unlucky enough to have an extremely preterm baby … most parents choose trying to save the baby because they have not had time to think what life would be like caring for a moderately or severely disabled child. (Neonatal nurse) If they are healthy this wont be needed. Why upset the mum as she will think something is wrong … the woman at risk could be identified … and then educated (Midwife)

## Discussion

Given that accurate information is essential for collaborative decision making by parents and medical staff around the treatment for periviable babies this study demonstrates that there is greater pessimism about the outcomes of the most premature babies by all HCP groups than is indicated by the actual outcome figures. Information is also important for enabling parents of babies at older gestations who will still require tertiary level intensive care for their babies to understand the risks to their offspring, and at older gestations, HCP are more accurate in their knowledge. HCP who have the most contact with parents prior to delivery, are the least accurate in terms of both mortality and the risks of a poor outcome at the lowest gestations. This discrepancy is concerning, as proactive antenatal care improves neonatal outcome, and where the antenatal team disagrees with the neonatal team in the provision of care, the outcomes for the baby are seen to be worse [18]. Where active care is proposed, antenatal steroids, magnesium sulphate, and monitoring of the foetus may optimise the condition of the baby and reduce later morbidity, hence decisions often need to be made well before delivery where possible [6, 18].

It is possible that the information as understood by HCP’s is merely out of date, however, whilst survival data has improved with time, there have been only modest improvements in the rates of severe disability seen in some studies [8]. Even in previous decades, the perceptions found for survival and disability would have been unduly negative, reflecting survival rates found in the late 1990’s [23, 24]. Studies done in the mid 2000’s reflect improved survival rates for babies offered intensive care [25]. In the Australian context with both inborn and retrieved babies improved survival rates are seen from the early 2000’s [26]. Undue negativity may reflect a reluctance of some HCP to provide care for these babies. Previous studies have shown that pessimistic clinicians are less likely to intervene to provide intensive care for periviable babies [19]. Hospitals with more optimistic obstetric and neonatal trainees are known to have received training from hospitals who have higher rates of providing care at the lowest gestations, and are found to be more accurate in their outlook [27]. Higher rates of offering care leads to improved outcomes [27, 28] and in some studies this appears to be regardless of numbers of small babies being cared for [28]. Whilst the tertiary unit described is a smaller tertiary centre in Australia, it has a high rate of offering care to babies under 25 weeks gestation [21] with comparative survival rates, but with more positivity it is likely that the survival and long term outlook for these babies would improve.

Extremely preterm babies will remain in the neonatal intensive care for months before going home. Parents who have experienced neonatal intensive care have been shown to have high rates of anxiety, depression, stress and trauma [29, 30] which may result in poorer long term developmental outcomes for the child [29]. Parents tell us that they need hope and honesty to help sustain them through their neonatal stay [31]. Whilst the potential for an adverse outcome needs to be understood by parents depending on the evolution of events during the babys care, if parents have been given a very negative outlook for their baby, the realistic hope that the baby may be healthy is removed, and the parent will need to endure the invasive painful treatment of the baby without recognising that the suffering baby has a potentially good outcome.

Staff based at smaller centres were found to be more negative about survival below 26 weeks than the tertiary HCP, but there was no difference in their perceptions of rates of disability. The origins for this are unclear. This has been noted in the Australian context in previous studies [32]. Most HCP at all centres were negative about long term outcomes. The non-tertiary centres will deliver fewer babies at extreme prematurity as an attempt to transfer antenatally to tertiary centres is standard care. Where parents presenting to these centres discuss the prognosis for their extremely premature babies, a more negative impression for potential survival will already have been conveyed to parents prior to transfer, and may have led to less optimisation of the fetus for postnatal survival, such as the administration of steroids [21, 33] at the referring hospital. The parents, in turn will have a more negative outlook for the baby and this may influence their decision making. Work to improve the knowledge at referral centres may improve the wellbeing of the delivered baby as shown in the work by Morse [19].

Clinicians who are involved in the care of women prior to delivery are significantly more negative than those who care for the baby in the short and long term. This confirms previous work done and has previously been shown to adversely affect the antenatal care of the extremely preterm fetus [10, 18]. Clinicians caring for the woman presenting with complications will have earlier counselling encounters with families and their more negative knowledge may affect parental decision making. Further research may reveal the origins of the more negative opinions.

All clinicians would offer care for patients at significantly lower gestations than they would wish for themselves, which is not unexpected given their negative perceptions of outcome. This has been described previously in trainee doctors [27] and may reflect a respect for patient autonomy and acceptance that patients may make different choices to the clinician. Furthermore, HCP recognised that there were specific factors about each pregnancy which would alter their risk assessment for the baby, and hence influence whether they thought that intensive care should be provided. Both surgical congenital anomalies and trisomy 21 were recognised as negative factors for survival and neurodevelopment, however, emotional factors such as previous pregnancy loss or the presence of no live born children in the family would positively encourage resuscitation despite no evidence that the difficult previous history will improve the outlook for the pregnancy at risk.

The difficulty in predicting an outcome for an individual pregnancy from large epidemiological studies was reflected in several free text comments. Whilst statistics may be important to clinicians, these reflections of uncertainty may be important factors for parents to understand. In a pilot study of 15 clinicians giving antenatal counselling, Prentice et al. [34] showed that most interactions involved the imparting of statistics and information only (60%) and eliciting parental preferences or engaging in deliberation was less frequent (20%). The nuance of the statistics and uncertainty with their application is unlikely to form part of this type of counselling. Previous studies have demonstrated that parents of extremely premature babies perceive the risk of death as more important than the risk of disability for a baby when a decision is made to resuscitate occurs [35–38]. HCP in these studies felt that the risk of severe disability was more important. Where death usually occurs in extreme prematurity, it is usually in the first days following delivery, so the uncertainty primarily affects the prognosis for disability, and this should be a part of counselling for decision making. Our study suggests that parents in North Queensland will receive a negative message about survival at gestations below 28 weeks, and rates of severe disability at the earliest gestations. At the earliest gestations, intact survival is similarly underestimated.

Most HCP recognise a need to support autonomy in parental decision making. However, this attitude was not consistently reflected in the answers to the range of questions asked. Where parents wanted intensive care provision for their baby but the clinician did not feel that it was in the best interests of the baby, 69.1% of respondents said that this care should be provided whilst 21% said that it should not. However, where parents did not want intensive care for the baby, but the clinician did, 37.5% would follow the parental request, but 40.6% would provide resuscitation despite this preference. Below 25 weeks, over half respondent felt that parents could be the sole decision makers, but 64.6% also said that clinicians could disregard parental choice at this gestation. It seems recognised that risks of death and disability decrease with increasing gestational age, but specific gestational cut offs are relatively artificial. Ethical dilemmas in the relative roles of parents and clinicians are reflected in these findings, with a range of opinions from complete parental autonomy to decline intensive care, even at gestations over 25 weeks, and those which deem that parents should not always be the final decision makers, even if intensive care then occurs for babies whose parents did not want this for their child. The data suggests that the trend is towards clinicians as the final arbiters of decisions. Further research could clarify the underpinnings of HCP beliefs.

Parental involvement in decision making can only be based on accurate information. Most guidelines currently in use in Australia, include parental discretion around the resuscitation of babies below 24 or 25 weeks gestation [3, 4, 39]. Despite the negativity of clinicians and guidelines discouraging the resuscitation of babies under 24 weeks, many of these babies are receiving intensive care in Australia and a recent review of the use of the consensus guidelines in New South Wales and Australian Capital Territory reflect that resuscitation at 23 and even 22 weeks regularly occurs [40]. In North Queensland, nearly all babies at 24 weeks gestation and nearly half of babies delivered at 23 weeks gestation receive tertiary intensive care, regardless of place of delivery [10]. With increasing parental autonomy, and parental requests for active care at gestations below 23 weeks, the perinatal community as a whole in Australia needs to be aware of improving outcomes and consider if the guidelines need modification to include clarity around resuscitation and provision of care at lower gestations.

There are some limitations to this study. It is a relatively small study based on a self-designed cross sectional survey from one area of Australia only which may limit the generalisability. However, local examination is important and the findings are consistent with those found in previous studies both historically and more recent. Another potential limitation is the use of the long term follow up data from the ANZNN. Follow up rates at the tertiary unit are relatively poor, and highest rates of follow up occurs for local babies within the immediate tertiary unit area and only one other regional centre where standardised tests are available.

The strength of this study is that it there was a good response rate, and that participation was invited from regional and remote centres where many patients initially presented with complications in their pregnancy. There are few studies which examine referring HCP knowledge. The survey also included staff whose contribution towards parental knowledge might previously have been ignored such as midwifery and neonatal nursing staff, as well as more junior obstetric staff. Midwifery and neonatal nursing staff will contribute to the parents’ perception of the long term with much closer daily contact whilst providing care both antenatally and postnatally and can influence the hope that parents need to cope with their neonatal experience. The inclusion of paediatricians who see these babies long term is also uncommon, but important as they will often have a long-lasting relationship with the children. A further strength of the study is that it has been done in an area with a high Indigenous population where Indigenous babies are over-represented on the neonatal unit. A strength of the study not reflected in most studies is the content analysis of the qualitative data. Qualitative data adds to the richness of the quantitative data in studies of knowledge and attitudes.

## Conclusion

Clinicians who work with pregnant patients need to give accurate information about the chances of survival and long term disability of babies who deliver at extreme prematurity if they wish to have collaborative decision making. This is most important for the senior clinician providing counselling but also important for other staff who may find themselves in a situation where their opinions will be revealed to the parents. Message framing will influence the parents decision making, but also their positivity during the neonatal unit stay. Enhanced positivity, without giving false reassurance, will improve parental experience of neonatal care and reduce the risk of poor mental health outcomes for the parent. Clinician bias needs to be explored to ascertain the source for undue negativity, and individual clinicians need to be responsible for ensuring that both their knowledge and biases are reflected upon. In the area studied, this study shows that improved education about prematurity is essential to improve the outcome of vulnerable babies and families. Units who offer intensive care for extremely preterm babies should be aware that accurate knowledge and positivity will improve outcomes. All tertiary hospitals providing neonatal intensive care need to regularly assess the adequacy of knowledge of their staff about extreme prematurity in this era of rapidly improving survival.

## Data Availability

Datasets used and analysed are not publically available due to the separation of the survey monkey results and analysis tool used, however this may be available from the corresponding author on reasonable request.
